# Characteristics and treatment outcomes of co-infected tuberculosis patients with human immunodeficiency virus in Southeast China, 2012–2021

**DOI:** 10.1186/s12879-023-08501-8

**Published:** 2023-08-10

**Authors:** Yinfa Zhou, Tao Li, Shufang Lin, Daiquan Chen, Yongcheng Du, Jiangfeng Chen, Kun Chen, Zhisong Dai

**Affiliations:** 1 Division of Tuberculosis Control and Prevention, Fujian CDC, Fuzhou, 350000 China; 2 National Center for Tuberculosis Control and Prevention, China CDC, Beijing, 100000 China

**Keywords:** Tuberculosis (TB), Human immunodeficiency virus (HIV), Characteristic, Drug resistance, Outcome, Risk factor

## Abstract

**Background:**

Tuberculosis (TB) is a chronic respiratory infection. Co-infection with human immunodeficiency virus (HIV) has been a significant obstacle to TB control. Insufficient attention has been given to TB/HIV, and more information is needed to address this issue. We conducted an observational study to investigate the epidemiological characteristics, treatment outcomes and its associated factors of HIV-positive TB patients in Southeast China.

**Methods:**

An observational study was conducted based on data collected directly from China National TB Surveillance System during 2012–2021. Epidemiological characteristics, drug resistance and outcomes were described as frequency (n) and percentage (%). Risk factors for unsuccessful outcomes were determined using univariate (chi-squared) and multivariate logistic regression analysis.

**Results:**

A total of 347 TB/HIV cases were included, and the proportion of HIV-positive cases among all TB cases increased significantly from 0.06% to 2012 to 0.40% in 2021. The majority of cases were males (86.5%), non-local household registers (139, 40.1%), farmers or workers (179, 51.6%), and aged 40–59 (142, 40.9%). Of 347 cases, 290 (83.6%) had pulmonary TB (PTB), 10 (2.9%) had extra pulmonary TB (EPTB) and 47(13.5%) had both PTB and EPTB. A total A total of 258 (74.4%) were HIV positive prior to TB diagnosis. 8.0% (4/50) of cases were resistant to rifampicin (RIF) and 274 patients (83.8%) had successful outcomes. Being non-local (*AOR* = 2.193, *95% CI* = 1.196–4.022, *P* = 0.011) and diagnosed HIV infection after TB (*AOR* = 2.365, *95% CI* = 1.263–4.430, *P* = 0.007) were independent risk factors for unsuccessful outcomes of anti-TB treatment.

**Conclusion:**

During 2012–2021, the proportion of HIV-positive cases among all TB cases increased significantly in Southeast China. HIV-positive TB patients were significantly more likely to develop resistance to RIF and INH and unsuccessful anti-TB treatment. Non-local registration and becoming HIV positive after TB diagnosis were independent risk factors associated with unsuccessful outcomes.

## Introduction

Tuberculosis (TB) is a chronic respiratory infection caused by *Mycobacterium tuberculosis*. Despite the availability of effective diagnosis and treatment, TB remains a threat to public health. Because of immunosuppression, co-infection with HIV is the risk factor for TB infection and the progression of latent TB infection. In addition, the lack of effective control measures for co-infection with HIV can lead to adverse outcomes of TB treatment, including death [1]. Co-infection with human immunodeficiency virus (HIV) has been identified as one of the most serious barriers to TB control [2]. To meet the ambitious goal of ending TB by 2035, TB/HIV control should be a crucial component.

Globally, in 2020, an estimated 8% of the 9900,000 TB patients (788,000) were HIV-positive, and there were 214,000 deaths among HIV-positive TB patients (up from 209,000 in 2019) [3]. China is one of the 30 high-burden countries in each of the three lists for TB (ranking second in the world in terms of cases), HIV-associated TB (TB/HIV) and multi-drug or rifampin resistant tuberculosis (MDR/RR-TB) [1]. In 2019, a total of 5,046 (1.1%) HIV-associated TB cases were registered in China. In contrast to TB patients (decreased at a rate of 2.2% per year), the number of TB/HIV patients is increasing, and the burden of TB/HIV co-infections increased by nearly 75% during 2015–2019. Rising mortality and adverse treatment outcomes among TB/HIV patients are also major concerns [4]. Fujian Province, Southeast China, has a low incidence of both HIV/AIDS and TB [5, 6]. However, the number of TB and HIV/AIDS cases reported in Fujian Province ranked third and fifth, respectively, among all reported infectious diseases groups stipulated by Chinese law [7]. To date, limited information on TB/HIV has been available in previous studies, insufficient to meet the End TB Strategy targets set for 2030 and 2035.

In this report, we analyzed TB surveillance data to examine epidemiological characteristics, treatment outcomes and its associated factors of HIV-positive TB patients in Fujian Province, Southeast China. Our study aims to complement the insufficient focus on TB/HIV and provide basic evidence for the prevention and control of TB/HIV co-infection.

## Methods

### Study design and data sources

This was an observational study based on data collected directly from China National TB Surveillance System. The research population consisted of notified TB patients in Fujian Province, Southeast China, during 2012–2021. The basic data about the patients and details were obtained from the patient’s medical records in the system. Population data were collected from the Yearbook of Fujian Provincial Bureau of Statistics.

Fujian Province, a southeastern coastal province of China, has a diverse distribution of coastal areas, encompassing 9 cities, Fuzhou, Putian, Quanzhou, Xiamen, Zhangzhou, Ningde, Nanpin, Sanming and Longyan. The 2021 population Census puts Fujian’s population at 415,40086.

### Study variables and definitions

Socio-demographic and clinical variables extracted from the medical records of TB patients in Fujian Province, including gender (male and female), age, occupation, household registration (local and non-local), HIV status, etiological examination results, time of HIV and TB diagnosis, sites of TB, history of TB treatment and outcomes. Patient addresses registered in the system were also collected.

Patients with pulmonary tuberculosis (PTB) and extra pulmonary tuberculosis (EPTB) were enrolled in the study. The mode of diagnosis of EPTB in an HIV positive individual is similar to that of PTB. It involves a thorough evaluation of clinical symptoms, X-ray findings, tuberculin and serological tests, bacteriological and pathological assessments, as well as other relevant test results. HIV status of known or newly confirmed HIV-positive TB patients was classified as TB/HIV co-infection. Patients with bacteriologically confirmed TB were diagnosed based on positive results from bacteriological tests such as smear microscopy, rapid molecular testing, or culture. Those without positive bacterial test results were classified as clinically diagnosed TB, determined through a thorough assessment of the patient’s clinical symptoms, X-ray findings, and other relevant test results. Positive specimens included sputum, bronchial lavage, and gastric aspiration. Anti-TB treatment outcomes were classified as cure, treatment completed, failure, death, or loss of follow-up according to the Technical Specification for Tuberculosis Prevention and Control in China.The outcomes of cured and treatment completed were collectively grouped as successful outcomes, whereas death, treatment failure and lost to follow-up were collectively grouped as unsuccessful outcomes. Patients transferred to MDR/RR-TB were removed from the outcome cohort.

### Bacteriology, drug susceptibility testing (DST)

All TB/HIV co-infection patients in this study were initially evaluated with three sputum specimens (spot, night, and morning) for fast acid bacilli by direct sputum smear microscopy using Ziehl–Neelsen stain or Xpert MTB/RIF (Cepheid, Sunnyvale, CA). Positive specimens were digested with an equal volume of 4% NaOH for 15 min, and then 0.1 mL of decontaminated specimens were inoculated on acidic Löwenstein-Jensen (L-J) medium for culture. Culture-positive samples were sent to the municipal TB laboratory for bacterial species identification and DST. DST was performed using the WHO-endorsed proportional L-J assay at the following concentrations: rifampicin (1 µg/mL) and isoniazid (0.2 µg/mL). The H37Rv MTB strain was tested with each batch of tests maintaining quality control [8, 9].

### Data statistics

All statistical analyses were performed using SPSS Statistics software version 24. Age was reported as continuous data with mean and standard deviation (*SD*) as it was normally distributed. Categorical data, such as gender, age groups, occupation, household registration, etiological examination results, time of diagnosis of HIV and TB, sites of TB, and history of anti-TB therapy were reported as frequency (n) and percentage (%). Univariate analysis (Chi-square test) was conducted to identify any statistically significant associations between explanatory variables and outcome variable (anti-TB treatment outcomes). All variables with *P* ≤ 0.20 in the univariate analysis were included in the multivariate logistic regression analysis. The final multivariate analysis model was constructed using the backward elimination approach. The crude odds ratio (*OR*) and adjusted odds ratio (*AOR*) were calculated during univariate and multivariate analysis, respectively, with 95% confidence intervals (*95% CI*). The significance level was set at *P* < 0.05.

## Results

### General trends, 2012–2021

During 2012–2021, a total of 160,309 TB patients were notified and monitored in Fujian Province, Southeast China. Both the absolute number of TB patients and cases per 100,000 population were steadily declining each year. The per 100,000 population of TB cases decreased significantly from 49.28 to 2012 to 32.43 in 2021(*χ2* = 2200.149, *P* < 0.001) (Table 1).

**Table 1:** TB and TB/HIV notified in Fujian province, Southeast China, 2012–2021

year	case of TB	per 100,000 population of TB	case of TB/HIV	% HIV-positive among all TB cases
2012	18,648	49.28	12	0.06
2013	17,616	45.86	14	0.08
2014	17,355	44.67	18	0.10
2015	16,515	41.86	26	0.16
2016	15,804	39.67	31	0.20
2017	14,668	36.52	40	0.27
2018	16,193	39.84	50	0.31
2019	15,654	38.14	45	0.29
2020	14,361	34.71	57	0.40
2021	13,495	32.43	54	0.40
Total	160,309	40.16	347	0.22

**Fig. 1:**
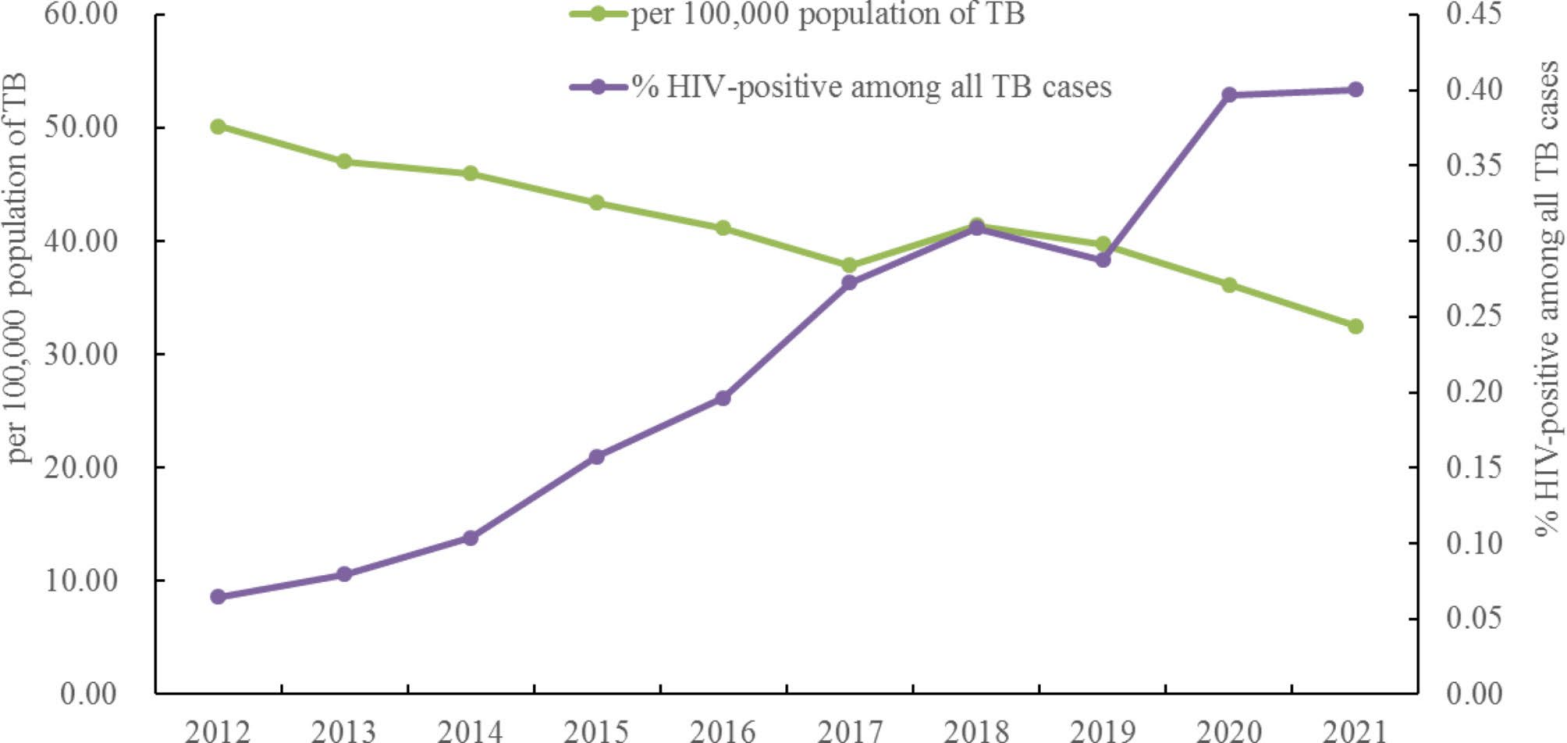
Per 100,000 population of TB cases and proportion of HIV-positive among all TB cases, by year, Fujian province, Southeast China, 2012–2021

Of these, 347 were classified as HIV positive. The number of TB/HIV co-infected patients was small compared to the number of TB cases without notification of HIV infection and represented only 0.22% of TB cases during the study period. However, in contrast to the population of 100,000 TB cases, the proportion of HIV-positive cases among all TB cases increased significantly from 0.06% to 2012 to 0.40% in 2021(*χ2* = 99.939, *P* < 0.001) (Fig. 1).

### Geographic distribution

Table 2 shows the number of reported HIV-positive TB and TB in 9 cities in Fujian Province. The geographic distribution of HIV-positive TB and TB is different in Fujian Province. The highest number of TB cases were found in Quanzhou City, Fuzhou City and Nanpin City. However, the highest HIV prevalence among TB cases was found in Nanpin City (0.65%), Longyan City (0.33%) and Fuzhou City (0.23%). HIV prevalence in TB cases was significantly different among 9 cities. (*χ2* = 132.339, *P* < 0.001) (Table 2).

**Table 2:** HIV prevalence within TB cases by city, Fujian province, Southeast China, 2012–2021

City	number of TB	number of TB/HIV	%HIV-positive among all TB cases
Nanping	10,117	66	0.65
Longyan	10,317	34	0.33
Fuzhou	30,692	72	0.23
Quanzhou	40,767	94	0.23
Ningde	11,895	24	0.20
Zhangzhou	18,960	23	0.12
Xiamen	16,065	17	0.11
Putian	12,385	12	0.10
Sanming	9111	5	0.05

### Socio-demographic and clinical characteristics

There was a predominance of males (86.5%), with a male-female ratio of 6.4:1. The mean (SD) age of patients was 45.8 (15.3) years (range 13–83 years), and the majority of co-infections 142 (40.9%) were reported in people aged 40–59 years. In terms of occupations, the majority of patients were farmers or workers 179 (51.6%), while 110 (31.7%) were unemployed or retired, 26 (7.5%) were employed in enterprises or government, and 32 (9.2%) were in other occupations. Patients with local household registration 208 (59.9%) were more than non-local 139 (40.1%).

258(74.4%) patients were diagnosed with HIV infection when TB was diagnosed, and 89 (25.6%) were diagnosed with HIV infection after TB. In addition, most patients 335 (96.5) received anti-TB treatment for the first time, and the remaining 12 (3.5%) were treated more than once. Of 347 TB/HIV co-infection patients, only 132 (38.0%) TB were bacteriologically confirmed by rapid acid bacilli smear microscopy, culture or rapid molecular test specimens (sputum, bronchial lavage or gastric aspiration). The remaining 215 (62%) cases, including all EPTB cases, were diagnosed clinically without any positive bacterial test results. (Table 3).

**Table 3:** Outcomes of TB/ HIV in Fujian, Southeast China, 2012–2021

Outcomes	Numbers	Percentage(%)
**Successful**	274	83.8
Cured	88	26.9
Treatment completed	186	56.9
**Unsuccessful**	53	16.2
Death	22	6.7
Lost to follow-up	31	9.5
**Total**	327	100.0

### Site of TB

According the site of TB, there are 290(83.6%) cases categorized as PTB, 10(2.9%) categorized as EPTB and 47(13.5%) had both PTB and EPTB among all 347 TB/HIV co-infected cases. Of the 57 patients with EPTB (including those who also had PTB), 44 (87.2%) were diagnosed at one site, and 13 (22.8%) at more than one site. Preilection sites were pleural 33 (57.9%), lymphatic 14 (24.6%) and meningeal 7 (12.3%).

### Drug resistance

Since 2018, we have only just begun to gradually promote drug resistance screening for all patients with bacteriologically confirmed TB. Therefore, DST results for TB/HIV patients in this study were rarely obtained. Results of RIF DST were obtained in 50 HIV-positive TB patients. Of these, 4 (8.0%) showed resistance to RIF as a result of DST. In addition, 5 (15.6%) cases were found to be resistant to INH in 32 TB/HIV patients conducted by INH DST. 3 (75%) rifampin-resistant (RR) TB/HIV cases were simultaneously resistant to INH, known as MDR-TB (Table 3).

**Table 4:** The results of RIF and INH DST of TB/HIV, Fujian, Southeast China, 2012–2021

INH	RIF		Total
	Resistive	Sensitive	
Resistive	3	2	5
Sensitive	1	26	27
no result	0	18	18
Total	4	46	50

### Outcomes and factors

Treatment outcomes for 331 TB/HIV patients were evaluated. Of these, 4 (1.2%) patients were transferred to MDR/RR TB treatment. Therefore, only 327 patients were included in the outcome cohort for analysis. Results classified as successful, including treatment completed (186) and cured (88), were 274(83.8%). The lower treatment success rate was due to the higher proportion of loss of follow-up 31 (9.5%) and deaths 22 (6.7%). (Table 4).

Univariate analysis found that household registration and time of HIV and TB diagnosis were strongly associated with unsuccessful outcomes of anti-TB treatment (*P* < 0.05). Compared to TB/HIV patients with local household registration, the odds of unsuccessful treatment were more than 2 times higher in non-local patients(*OR* = 2.270, *95% CI* = 1.250–4.120, *P* = 0.006). The odds of unsuccessful treatment in patients diagnosed with HIV infection after TB were over 2.6 times higher than those in patients diagnosed with HIV infection before TB.(*OR* = 2.377, *95% CI* = 1.286–4.395, *P* = 0.005) (Table 5).

**Table 5:** Outcomes of HIV-positive PTB cases by selected characteristics, Fujian, Southeast China, 2012–2021

Characteristics	Total	Outcomes		Crude OR (95% CI)	P-value
		successful	unsuccessful		
**Sex**					
male	300	237	46	1	
female	47	37	7	0.975(0.409–2.320)	0.954
**Age group**					
~ 39	128	108	18	1	
40 ~ 59	142	106	21	0.714(0.332–1.537)	0.39
60~	77	60	14	0.849(0.402–1.792)	0.668
**Occupational category**					
Farmers and workers	179	147	20	0.735(0.254–2.126)	0.57
corporate and government employees	26	22	4	0.982(0.235–4.104)	0.98
unemployed and retired	110	78	24	1.662(0.577–4.787)	0.347
Other job	32	27	5	1	
**Household registration**					
local	208	174	23	1	
Non-local	139	100	30	2.270(1.250–4.120)	0.006
**Diagnosis**	0				
bacteriological	132	100	24	1	
clinical	215	174	29	0.694(0.383–1.258)	0.227
**Anti-TB therapy history**					
Yes	12	8	4	1	
No	335	266	49	2.714(0.787–9.364)	0.101
**Time of HIV and TB diagnosis**					
HIV earlier	258	211	30	1	
TB earlier	89	63	23	2.377(1.286–4.395)	0.005
**Site of TB**					
PTB	290	230	43	1	
PTB and EPTB	47	36	9	1.496(0.182–12.265)	0.708
EPTB	10	8	1	2.000(0.221–18.112)	0.538
**Total**	347	274	53		

In addition to household registration and time of HIV and TB diagnosis, history of anti-TB therapy with *P* ≤ 0.20 in the univariate analysis was included in the multivariate logistic regression analysis. Multivariate analysis revealed that household registration and time of HIV and TB diagnosis were still independent factors associated with unsuccessful outcomes of anti-TB treatment. After adjusting for other variables, non-local TB/HIV patients had almost twice the odds of unsuccessful treatment (AOR = 2.193, *95% CI* = 1.196–4.022, *P* = 0.011), compared to patients with local household registration. The odds of unsuccessful anti-TB treatment outcomes in patients diagnosed with HIV infection after TB were more than twice that of patients diagnosed with HIV infection before TB (*AOR* = 2.365, *95% CI* = 1.263–4.430, *P* = 0.007). (Table 6).

**Table 6:** Final model of multivariate analysis showing the risk factors of unsuccessful outcome

Characteristics	Successful	Unsuccessful	AOR	95% CI	p-Value
**Household register**					
local	174	23	1		
Non-local	100	30	2.193	1.196–4.022	0.011
**Anti-TB therapy history**					
Yes	8	4	1		
No	266	49	3.152	0.808–11.184	0.076
**Time of HIV and TB diagnosis**					
HIV earlier	211	30	1		
TB earlier	63	23	2.365	1.263–4.430	0.007

## Discussion

This study shows that the incidence of TB decreased year by year in Fujian Province during 2012–2021. However, the current annual rate of reduction was still far from the End TB Strategy targets set for 2030 and 2035. And it cannot be met without intensified research and innovation [3]. Therefore, more efforts will now be needed to achieve an even faster rate of decline in TB incidence. Although the proportion of HIV positivity among TB cases in Fujian Province was lower than that of other nations and other provinces in China [4, 10, 11], similar to Japan [12], it has shown an increasing trend in recent years, perhaps due to improved case-finding and reduced TB cases. In addition, the geographic distribution of HIV-positive TB and TB is different in Fujian Province. And HIV prevalence in TB cases was significantly different among 9 cities. Therefore, HIV co-infection control should be one of the most important components of the TB control program in Fujian Province.

Our study demonstrated socio-demographic characteristics of 347 cases diagnosed with TB/HIV co-infection. Results showed that the majority of the participants in this study were male, 40–59 years of age, local household registers, farmers and workers. These groups have more social and economic activities, as well as risk factors associated with TB and HIV prevalence [13]. This was consistent with the socio-demographic characteristics of notified TB patients in Fujian Province, reported in the previous study [14]. This means that co-infection with HIV did not change the socio-demographic characteristics of TB. We should pay more attention to these groups in TB control, especially in TB/HIV co-infection control.

It should also be noted that TB/HIV patients without bacteriological confirmation were more than half in this study, and none of the patients with EPTB were bacteriologically confirmed. This would make TB/HIV diagnosis challenging and increase the likelihood of avoiding or delaying diagnosis, and more likely to spread the disease [15]. Although a lower burden of mycobacterial organisms and a lower likelihood of HIV-induced cavitation due to immunologic suppression in TB/HIV patients may be one reason, poor performance of standard diagnostic tools is also a significant barrier to TB diagnosis in HIV-positive patients. More sensitive tools (rapid molecular testing or culture) are needed to be developed and implemented to diagnose TB in HIV-positive patients.

Because of the large proportion of INH resistance in RIF-resistant strains, RIF resistance is seen as a proxy for MDR-TB [16]. In this study, we reported that 8.0% were found to be resistant to RIF and 15.6% to INH in patients undergoing DST. Both RIF - resistant and INH - resistant rates were higher in TB patients with HIV- positive TB patients than in all TB cases (5.9% and 8.3%) reported in the previous study on drug-resistant surveillance in Fujian Province [17]. It suggests that TB patients who are co-infected with HIV are more likely to be resistant to RIF and INH. Non-compliance with tuberculosis medication regimes, poor autoimmune immunity, and non-adherence to treatment due to adverse reactions may be material causes of RIF and INH resistance [1]. It should be noted that the relative low proportion of drug resistance patients in our analysis due to the late development of drug resistance screening may not well present the real condition of TB/HIV patient’s drug resistant status. We will conduct further research based on enhanced drug resistance screening in the coming years.

Globally, in 2019, treatment success rates remained lower among TB patients living with HIV (77%) [2]. There is no significant difference with the therapeutic regimen between HIV positive and negative TB in Fujian Province, but ART is required in conjunction with TB treatment. Treatment success rates for TB/HIV patients in Fujian Province (83.8%) were higher than in the world, but lower than in the country (87.6%) [4]. Consistent with previous reports [18, 19], we found that HIV-positive TB were more likely to develop unsuccessful anti-TB treatment outcomes, due to more deaths and lost to follow-up.

Non-local household registration and diagnosis of HIV infection after TB were identified as independent risk factors for unsuccessful TB treatment in the final model. Comparing the local population with TB/HIV, non-local patients were more likely to develop unsuccessful treatment outcomes. Non-local household registers may have no fixed residence, a wide range of activities and poor economic conditions, and this will lead to instability in access to treatment. Patients diagnosed with HIV infection after TB were also more likely to have unsuccessful outcomes, possibly due to immune destruction caused by HIV co-infection with delayed antiretroviral therapy (ART). Therefore, early diagnosis of TB/HIV with two-way screening, including HIV antibodies in TB patients and active TB in HIV/AIDS patients, as well as timely ART and anti-TB treatment, should be introduced to reduce the risk of unsuccessful anti-TB therapy, as well as health education and treatment management. Furthermore, accurate TB diagnoses are essential. To avoid unsuccessful treatment outcome caused by incorrect diagnoses, we emphasize the implementation of stringent measures to ensure precise tuberculosis diagnoses in Fujian Province’s TB control program.

This study had several limitations. First, this study did not assess patients’ ART. Several studies have shown that ART status is strongly associated with the outcomes of anti-TB treatment [20, 21]. Second, due to the data source, limited variables were analyzed in this study.

## Conclusions

Although co-infection with HIV did not change the epidemiological characteristics of TB, the increasing proportion of HIV positivity among TB patients and the different geographic distribution in 9 cities shown in this study require ongoing monitoring. Also, HIV-positive TB is more likely to develop into RIF resistant and unsuccessful anti-TB treatment. Non-local registration and HIV diagnosis after TB were independent risk factors associated with unsuccessful outcomes. Therefore, to prevent the incidence of TB/HIV, especially TB/HIV with RIF resistance, we need to increase efforts to detect and treat both HIV infection and TB, provide preventive treatment for latent TB infection and sufficient treatment of TB to reduce the burden of HIV-associated TB, as well as to prevent further TB transmission.

## Data Availability

Data Availability Statement: All relevant data in this study is freely available in the manuscript as submitted. And the data are included in the tables and results described in the manuscript. Datasetare is available from the corresponding author upon reasonable request in the following email: simonwind100@126.com.
